# Facial thread lifting with suture suspension^☆^

**DOI:** 10.1016/j.bjorl.2017.03.015

**Published:** 2017-05-09

**Authors:** Joana de Pinho Tavares, Carlos Augusto Costa Pires Oliveira, Rodolfo Prado Torres, Fayez Bahmad

**Affiliations:** a Universidade de Brasília, Programa de Pós-Graduação em Ciências da Saúde e Departamento de Otorrinolaringologia, Brasília, DF, Brazil; b Universidade de Brasília, Escola de Medicina, Brasília, DF, Brazil; c Universidade de Brasília, Faculdade de Ciências da Saúde, Brasília, DF, Brazil

**Keywords:** Rhytidoplasty, Lifting, Sutures, Ritidoplastia, Rejuvenescimento, Suturas

## Abstract

**Introduction:**

The increased interest in minimally-invasive treatments, such as the thread lifting, with lower risk of complications, minimum length of time away from work and effectiveness in correcting ptosis and aging characteristics has led many specialists to adopt this technique, but many doubts about its safety and effectiveness still limit its overall use.

**Objective:**

To analyze data published in the literature on the durability of results, their effectiveness, safety, and risk of serious adverse events associated with procedures using several types of threading sutures.

**Methods:**

Literature review using the key words “thread lift”, “barbed suture”, “suture suspension” and “APTOS”. Due to the scarcity of literature, recent reports of facial lifting using threads were also selected, complemented with bibliographical references.

**Result:**

The first outcomes of facial lifting with barbed sutures remain inconclusive. Adverse events may occur, although they are mostly minor, self-limiting, and short-lived. The data on the maximum effect of the correction, the durability of results, and the consequences of the long-term suture stay are yet to be clarified.

**Conclusion:**

Interest in thread lifting is currently high, but this review suggests that it should not yet be adopted as an alternative to rhytidectomy.

## Resumo

**Introdução:**

O maior interesse por tratamentos minimamente invasivos, como os fios de sustentação, com menor risco de complicações, mínimo tempo de afastamento das atividades laborais e eficazes em corrigir a ptose e as rítides características do envelhecimento fez muitos especialistas adotarem esta técnica, porém muitas dúvidas sobre sua segurança e eficácia limitam sua adoção de forma geral.

**Objetivo:**

Analisar dados publicados na literatura sobre longevidade dos resultados, sua eficácia, segurança e risco de eventos adversos graves associados aos procedimentos com vários tipos de sutura.

**Método:**

Revisão de literatura utilizando palavras-chave “*thread lift*”, “*barbed suture*”, “*suture suspension*” e “APTOS”. Devido à escassez de literatura, foram também selecionados relatos recentes de rejuvenescimento facial com uso de fios, complementados com trabalhos das referências bibliográficos.

**Resultado:**

Os primeiros resultados da suspensão facial com fios com garras permanecem inconclusivos. Os eventos adversos podem ocorrer, embora em sua maioria sejam pequenos, autolimitados e de curta duração. Os dados sobre o efeito máximo da correção, a longevidade dos resultados, e as consequências da permanência dos fios em longo prazo não são claros.

**Conclusão:**

O interesse pelo *lifting* com fios está em alta no momento, mas esta revisão sugere que ele não deve ser apresentado como alternativa a uma ritidoplastia.

## Introduction

Since the earliest reports of facial surgical rejuvenation by Miller and Kolle,^1^, ^2^ more durable and less invasive means of rejuvenating the face have been sought. A better understanding of the alterations that occur to facial soft tissues, leading to an aged appearance, has allowed the development of new techniques to deal with the specific anatomy of facial aging.

Following that, rejuvenation techniques developed from skin-tension-only procedures, focussing on a variety of dissection and fixation planes: subcutaneous, sub-SMAS (superficial muscular aponeurotic system) and subperiosteal.^3^ It is accepted today that, regardless of the technique used, any facelift procedure should consider the fact that the deeper tissues must be repositioned or filled before the skin is pulled and resected.^4^ For all these techniques, soft tissue suspension with absorbable or nonabsorbable sutures is essential.^3^ Surgical lifting with redundant skin excision has been the standard lifting procedure for decades, offering a radical and, often stigmatizing, surgical solution.^5^, ^6^ Recent understanding and appreciation of the vectors that must be applied to achieve optimum tissue elevation has improved the outcomes, by repositioning ptotic soft tissues into a more anatomical vertical direction.^3^ Surgical interventions, however, are accompanied by possible complications such as infection, skin necrosis, hematomas, seromas and injury to the frontal and marginal branches of the facial nerve, in addition to the risks involved in general anesthesia or even conscious sedation.^6^ They are also associated with visible scars and prolonged recovery.^5^, ^6^, ^7^ In many situations, patients prefer minimally-invasive procedures and are willing to accept a more modest degree of esthetic improvement in return for decreased morbidity and rapid healing.^6^

Non-surgical rejuvenation by volumetric increase with several types of interventions, including injections with a variety of gels or fat, added the “third dimension” to facial rejuvenation.^3^, ^5^, ^8^ However, while good results have been reported and can be achieved when performed by experts who have an artistic touch, the use of fillers may result in increased facial volume with unnatural contours, visibly shifting the gravity center of the face to its lower third.^5^ Ablative or non-ablative resurfacing techniques allow the improvement of the skin surface, but do not adequately lift the underlying ptotic tissues, an important step in achieving a younger appearance.^5^

The use of threads for facelift procedures is not a new idea.^9^, ^10^ This procedure involves the passage of sutures under the skin of the face and neck to compensate for sagging and flaccid tissues, avoiding large incisions and greatly reducing recovery time.^9^ Although it is a much-discussed procedure in the lay press, there is little information in the specialized medical literature about its safety, efficacy, durability, and possible complications.^9^

## Objective

To analyze findings in the literature on the results of studies with suture suspension in relation to their efficacy, durability, safety of permanent implantation of barbed sutures and risk of serious adverse events associated with the procedure.

## Methods

A search was carried out in the PUBMED database for studies using the keywords “thread lift”, “barbed suture”, “suture suspension” and “APTOS”; recent relevant reports on facial rejuvenation using thread were identified and selected. There were no exclusion criteria, since the objective was not to evaluate the validity of the presented evidence, but the general points of view expressed in the literature. These reports were complemented with studies identified in the bibliographic reference lists of the assessed publications.

### Tissue suspension with suture

The use of absorbable or non-absorbable sutures has long been the basis for repositioning and supporting subcutaneous tissues.^11^ Suspension techniques together with traditional rhytidectomy incisions are generally used to achieve better facial rejuvenation results. Skin and SMAS suspension, or deep subperiosteal suspension, can be performed with autologous tissue, such as tendons or fascia, or prosthetic materials, including sutures, slings, or mesh.^6^, ^9^ Several types of suture material have been used for this purpose, particularly polytetrafluoroethylene (Gore-Tex™), polyglactin (Vicryl™) and polypropylene.^9^ Among the many suspension techniques, two general concepts of facial rejuvenation are currently being developed.^12^ The first is subcutaneous suspension using SMAS as the fixation basis, with tissue elevation using posterolateral vectors, and the second is based on the subperiosteal detachment and en-bloc repositioning of all structures using purely vertical vectors.^12^ Suspension techniques have also been incorporated into minimally-invasive procedures of the middle and lower facial thirds using the endoscope. Thus, it is possible to reposition and anchor the facial soft tissues to the temporal fascia or the periosteum through minimal incisions.^13^

Simple transfixation of smooth threads through the skin can be a suitable option to elevate the eyebrows or the middle third of the face with low morbidity.^7^, ^14^ Several techniques have been described that achieve a subtle facial lifting with the use of suture threads that hold the area of the face to be suspended, ultimately fixing it to the scalp (Curl Lift, Arch Lift).^15^ The results of these procedures may be unpredictable. The threads tend to easily cut the tissues and the wrinkles and folds of the skin tend to disappear along with the results.^7^, ^14^

### Barbed sutures

With the advent of polypropylene barbed sutures, the capacity of the sutures to withstand loads increased very much.^9^ The first to mention the concept of barbed sutures was Alcamo in 1964,^7^ followed by Fukuda in 1984 and Ruff in 1994.^16^ These pioneers used the barbed sutures to close wounds without the need for knots. However, they did not anticipate their esthetic applications.^7^ The suture they described had barbs placed sequentially along its length. The barbs changed direction at a point near the center of the thread to create a mirror image of the barbs in the opposite direction. When wrapped around the tissues, one extremity anchored the other and closed the wound or moved the tissues toward the center of the thread, creating new gradients of tension and compression.^16^, ^17^

Tissue suspension with barbed sutures was first reported in the late 1980s by Russian authors Sulamanidze et al.^5^, ^18^ With little or no soft tissue dissection, the sutures are placed in the subcutaneous tissue.^3^, ^18^ Both the design and the way they were placed have changed since their introduction. The original APTOS (Anti-Ptosis Suture) was a multi-toothed thread that was later modified to become bidirectional, with the barbs directed to attach the tissues to the central region of the thread, without the need for anchoring at the extremities. It was subsequently redesigned to allow one-way traction and suspension.^18^ With the one-way barbs, the fixation methods were once again modified.^3^, ^18^ Although several techniques have been used over the years, all of them involve the interposition of the soft tissues to the suture barbs, with consequent triggering of inflammatory response and production of fibrosis around them.^19^

The latest generation of barbed sutures for soft tissue support is available as both non-absorbable and absorbable material with several lengths and different types of inlaid needles.^3^ The varied current presentations allow different applications, including self-fixation, curved passages that embrace the ptotic tissue, loops, and fixation of the two extremities of the thread at a single point, with anchoring at the temporal fascia or other deep points.^7^, ^15^, ^19^

The mono-filament, double-convergence polypropylene threads used in Brazil, known as Beramendi or Russian threads, are approved by ANVISA, but not by the FDA.^18^, ^20^ In general, permanent strands are approved for correction of facial and cervical ptosis, while absorbable threads are approved only for soft tissue approach and suturing of incisions.^17^ The most common absorbent material used to produce the threads is polydioxanone (PDO), a polymer that is gradually hydrolyzed.^16^

Barbed sutures can be applied to both the forehead and the middle and lower thirds of the face, as well as the neck. Regardless of where the threads are used on the face, incisions, however small, should be made in locations where they can be camouflaged, followed by dissection of the subcutaneous tissue, passage of the threads and their proximal anchoring. Finally, the soft tissues should be shaped so that a smooth and harmonious contour is achieved.^3^

## Result

### Evidence of results and long-term efficacy

It is important to emphasize that long-term studies and reviews on the duration of results and the satisfaction of patients undergoing facial rejuvenation procedures with support sutures are rare.^6^, ^21^ The few existing clinical studies have a level of evidence III, at the most.^9^

Sulamanidze et al. stated that elevating facial tissues with APTOS is simple, effective, and fast, and it prevents scarring and the need for implants.^18^ The authors also stated that both immediate and late outcomes are good and long-lasting (follow-up from 2 months to 2.5 years).^18^, ^22^ Lycka et al. also reported good results in a series of 350 patients in a retrospective study based on “patient interviews and surgeon observation.”^23^ They also defended the use of threads in replacing traditional surgical interventions citing minimal complications and high patient satisfaction.^23^, ^24^ For them, APTOS would be a safer alternative to the current facelifts, suggesting that the well-positioned barbed sutures have the effect of making the face look more harmonious and thinner.^7^ Regarding the duration of the results, 117 patients were followed up for a period of 12–24 months and maintained 70% of the initial correction, determined by blind photo evaluation.^23^ Others have found that facial tissues seem stronger, firmer and rejuvenated, and, more importantly, patients seem happy with the results, although very little persistent elevation can be observed.^24^ This reported improvement may have occurred due to the production of fibrotic tissue around the sutures, combined with the effect of soft tissue structuring also promoted by suturing. It is known that fibrosis increases the collagen matrix of the dermis and subcutaneous tissue.^7^

The enthusiasm for the so-called “thread lift” in treatment of the nasolabial, bucco-mentonian grooves, eyebrows, and platysmal bands has been disseminated by patients themselves.^19^, ^23^, ^24^ In the short term after the procedure (1–3 months), many state they seem to have undergone a “perfect face lift” and, like their doctors, are delighted with the quality of the results obtained.^6^ Published data from an anonymous satisfaction survey of 20 consecutively treated patients and based on the pre- and post-procedure photo evaluation of 7 patients by 7 independent dermatologists concluded that the unidirectional, permanent and anchored barbed sutures (Contour Threads^®^) placed in subcutaneous tissue can produce long-lasting ptotic facial tissue suspension.^6^ Based on these results, the hypothesis is that the correction lasts at least a year or a little longer than that.^6^ The thread advocates also mention the rapid recovery of their patients after the procedure.^22^ Although there are many advantages in using the barbed sutures for minimally-invasive facelift procedures,^6^ its long-term effectiveness may be disappointing, leading to a decline in its popularity.^24^ Overall, the results obtained are subtle and have a short duration.^11^

Some plastic surgeons have questioned the allegations of predictable, long-lasting, low-morbidity results with a minimal complication rate, made by thread use advocates.^22^ They argue that the results are disappointing and that tissue repositioning is not maintained in the long term, and threads can often be visible, extruded, broken, or traction lines can appear at rest or with facial mimicry.^11^ Despite reported cases of successful treatment of the mandibular line and neck,^11^ it can be observed that the skin of the lower third of the face tends to sag over time.^6^ Even when the techniques are modified to tie the fascia that covers the platysmal muscle, the results in the neck are still inferior to those observed in the malar region.^6^

### Complications of facial lifting with threads

Poor and short-term results may not be the only risks of this noninvasive facial rejuvenation procedure. Permanent insertion of a foreign body can cause unexpected reactions with considerable morbidity and negative esthetic effects, and even the most stable alloplastic material may fail and break down under repeated stress and flexion, such as occurs in the moving face.^11^ Reports on side effects of a wide variety of alloplastic materials applied to the face for this purpose have increased, and a more stringent monitoring of these effects is necessary.^19^, ^25^ Although immunological or chemical reactions to an inert implant are very unlikely, such foreign bodies can infect or fragment^25^ Because these procedures are commonly performed on relatively young patients and complete surgical suture removal is virtually impossible, residual fragments can remain for decades, worsening symptoms over time.^25^

There have been no major complications reported in most studies on the use of barbed sutures.^5^, ^8^, ^18^, ^23^ Minor and transient complications include facial asymmetry, bruising, erythema, hematoma, edema, and discomfort.^11^ Thread migration, extrusion, and scar formation at their sites of entry and exit are the late complications described.^11^, ^26^ Complications of APTOS were more recently reported in a series of 102 patients: 11 palpable thread tips with pain, 8 thread migrations, 5 infections or granulomas, and 5 skin irregularities.^7^, ^9^ More severe complications, such as Stensen's duct rupture, facial nerve injury, chronic foreign body sensation and scarring have been reported by other authors.^25^, ^26^

In addition to the previously reported transient bruising and edema, some patients may still have irregularities in the skin overlying the threads. Although transient, they may persist for days or weeks. The patient generally cannot return comfortably to his/her daily activities until the resolution of such irregularities. Thus, the time required for recovery after the “thread lifting” may be the same for the recovery after a rhytidoplasty.^24^ Additionally, the rate of surgical revision after the thread lifting procedures is high. In all, 11% of patients require removal of the threads because they are palpable, extruded, or due to patients’ dissatisfaction with their appearance.^24^

The data of the studies evaluated in this review are summarized in Table 1 below, which is an extended version of the table published by Villa et al. in their review.^9^

**Table 1:** Review of the series of cases of patients submitted to facelift with barbed sutures.

Author	Type of thread	No. of patients	Complications	Duration of follow-up	Assessment of results	Ideal patient
Lycka et al.,^23^	Aptos threads	350	47% bruising; 46% bleeding; 43% edema; 14% erythema; 14% discomfort; 3% visible threads; 3% asymmetry	117 patients followed for 12–24 months maintaining 70% of the initial correction; 96 patients followed for 36 months, maintaining 60% of the initial correction.	56.5% very good to excellent; 27.7% good; 13.4% fair; 2.4% unsatisfactory or poor	Young patients with many wrinkles or after rhytidectomy who desire mild to moderate improvement
Wu,^7^	Aptos threads	102	11% palpable thread tip with pain; 8% thread migration; 5% infection or granuloma; 5% skin retraction or irregularity	Not recorded	No objective elevation measurement	Not recorded
Sulamanidze et al.,^5^, ^18^	Aptos threads	157	14.6% skin retraction; 9.5% linear hemorrhage; 9.5% hypercorrection; 7.6% hypocorrection; 2.5% thread expulsion	2 months to 2.5 years	No objective measure of elevation; 12 cases required additional threads	Ptosis, face and neck flaccidity and poorly defined facial contours
Silva-Siwady et al.,^28^	Aptos threads	1	Migration and expulsion	28 days	No objective elevation measurement	Not recorded
Lee and Isse,^29^	Contour threads	44	20% sensitivity in the zygomatic arch; 10% hematoma; 3% bruises; 3% skin retraction	9 months (mean)	No objective elevation measurement	Patient with isolated malar fat ptosis and minimal loss of mandibular contour
Badin et al.,^20^	Beramendi threads	52	13.5% skin irregularities; 9.6% extrusion; hyperalgia 3.8%	18 months	No objective elevation measurement	Ptosis of the middle third of the face with palpable adipose tissue
Horne et al.,^30^	Contour threads	25	4% 50% loss of thread tensile strength after 11 weeks	12 weeks	No objective elevation measurement	Not recorded
Helling et al.,^25^	Not specified	4	1 wound secretion, local pressure, and asymmetry; 1 foreign body sensation and palpable thread; 1 facial paralysis; 1 skin irregularity	1 month, 1 year, soon after 5 months	No objective elevation measurement	Not recorded
Kaminer et al.,^6^	Contour threads	12	58% swelling and bruising; 36% thread visibility; 27% sensation of pinching; 25% ear numbness	6–16 months (mean 11.5 months)	Patient subjective satisfaction classification and independent evaluators of 1–10: mean of 6.9	Not recorded
Sardesai et al.,^24^	Not specified	75	23% patient dissatisfaction; 19% extrusion requiring procedure for thread excision or repair; 4% skin irregularities	Not recorded	No objective elevation measurement	Not recorded
Garvey et al.,^31^	Contour threads	72	31% revision surgery for cosmetic reasons (mean of 8.7 months later); 11% tip removal from palpable threads	Not recorded	42% needed a secondary thread procedure on average 8.4 months later	Not recorded
Gamboa et al.,^32^	Silhouette Lift	17	100% temporary edema; 6% submitted to resuspension 4 months later	9 months	90% satisfaction; 11% moderate improvement	Not recorded
Rachel et al.,^33^	Contour threads	29	69% of adverse events and 45% of early recurrence (6 months), 14% of which had recurrence within 8 weeks	1–25 months	No objective elevation measurement	Not recorded
Sulamanidze et al.,^34^	Aptos threads	6098	3% asymmetry; 2.8% contour irregularities; 2.7% ptosis recurrence; 1% skin retraction; 0.2% hematoma	Not recorded	No objective measurement. Results improved as thread quality and techniques improved in 12.5 years	Age younger than 50 with moderately flaccid cheeks
Savoia et al.,^35^	Happy Lift	37	62% mild bruising; 40% moderate erythema; 40% slight postoperative swelling; 25% mild bleeding; 6% asymmetry; 6% mild transient anesthesia	6 months	Rating: 1 excellent, 2 much better, 3 better, 4 unchanged, 5 worse. 89% satisfactory results (65% excellent and 24% good) and 11% unsatisfactory results	Moderate ptosis
Park et al.,^36^	Polypropylene developed by the authors	102	2% dissatisfaction, 1% skin irregularity, 1% weakness of the facial temporal branch	5–18 months	98.1% patient satisfaction. Surgeon's Satisfaction: 69.6% excellent; 7.5% good; Fair 2.9%	Not recorded
De Carolis et al.,^37^	Polypropylene developed by the authors	67	13.4% of thread visibility at movements; 1.5% hematoma; 1.5% facial paralysis	18 months	No objective elevation measurement	First signs of neck flaccidity or oblique cervical-mentonian angle
Han et al.,^38^	Reeborn	18	5.5% minimum irregularity; 5.5% palpable suture	12 months	Significant reduction of wrinkles according to Fitzpatrick's Modified Wrinkle Scales and Marionette Line grade 6 months: *p* < 0.0001 12 months: *p* < 0.0003 100% patient satisfaction	Moderate to severe wrinkles and visible marionette lines

## Discussion

The introduction of new procedures and technologies usually occurs in a cautious manner, with improved acceptance after initial skepticism.^25^ When these new techniques fail to deliver what they promise, or when better technologies emerge, they soon fall into oblivion.^24^ The use of thread lifting for face suspension is not a new idea and is now in its third decade of evolution.^27^ Barbed sutures have been presented as the method to achieve suspension and facial rejuvenation without surgery, earning the interest of patients and surgeons.^23^ The ideal patient is young, does not have many wrinkles or much redundant skin, or is a patient who underwent a rhytidoplasty, of which results still require a slight to moderate improvement.^23^ Threading is not indicated when there is significant photoaging or very prominent wrinkles.^23^ There is still no consensus on the number of threads to be used, or on how to best to position them.^23^ However, as the understanding of the vectors to be applied on the face has developed, new thread introduction patterns are being developed to produce better results. Possibly, the evolution of scientific studies and clinical experience will determine the best way to position the threads, their role when associated with other forms of facial lifting and their role as an option in facial rejuvenation.^23^

New techniques must be guaranteed to maintain the advances. The studies should include standardized and objective photographic analysis, with fixed intervals in the postoperative period, and double-blind evaluation. Patient groups matched for age, gender and skin characteristics or randomized in experimental and control groups should be made for future studies comparing the “thread facelift” with the standard facelift techniques. Laboratory and animal studies should assess the biomechanical and biochemical reactions of the threads in a biological environment.^9^ Finally, it should be assessed whether the best results are reproducible and relatively independent of the operator.^9^ Side effects, long-term results and complications should also be considered and scientifically reported.^26^ Other questions that require consideration are the effect of threads on facial mimicry and what happens to them with repeated facial movement. It should also be clarified how the complications can be solved and if the placement of the threads affects in some way or prevents the performance of other esthetic or reconstructive procedures in the future.^9^

Quick non-surgical procedures, often advertised as an alternative to rejuvenating surgeries, are in fashion. Despite the popularity of the “thread facelift”, it should not be presented as an alternative to surgical facelift and should be seen only as a temporary procedure that can be maintained until patient aging requires further approaches.^22^ Patients will be disappointed if they expect the thread facelift to show the same outcome as traditional facelift surgery.^22^ However, new variations of barbed threads are being developed and technological progress may bring useful novelties in the near future.^24^ For the time being, traditional surgery remains the gold standard for facial rejuvenation.^24^ Although it seems simple to suspend the ptotic tissues of the face as a puppet, deeper knowledge about the anatomy and physiology of aging underscores the need for the surgical approach to redistribute and suspend the different layers of the face through open or endoscopic access.^22^

## Conclusion

The interest in thread facelift is currently high and, although it still lacks more detailed studies, this review suggests that it should not be accepted as an alternative to a traditional rhytidectomy, since data on its indications, complications, efficacy and duration of results are still inconclusive.

## Conflicts of interest

The authors declare no conflicts of interest.
